# Towards a Standardized Antimicrobial Susceptibility Testing Method for *Mycoplasma hyorhinis*

**DOI:** 10.3390/microorganisms11040994

**Published:** 2023-04-11

**Authors:** Lisa Käbisch, Anne-Kathrin Schink, Doris Höltig, Joachim Spergser, Corinna Kehrenberg, Stefan Schwarz

**Affiliations:** 1 Institute of Microbiology and Epizootics, Centre for Infection Medicine, School of Veterinary Medicine, Freie Universität Berlin, 14163 Berlin, Germany; 2 Veterinary Centre for Resistance Research (TZR), Freie Universität Berlin, 14163 Berlin, Germany; 3 Institute for Veterinary Food Science, Department of Veterinary Medicine, Justus-Liebig-University Gießen, 35392 Gießen, Germany; 4 Division for Pigs, Farm Animal Clinic, School of Veterinary Medicine, Freie Universität Berlin, 14163 Berlin, Germany; 5 Institute of Microbiology, University of Veterinary Medicine, 1210 Vienna, Austria

**Keywords:** antimicrobial susceptibility testing (AST), broth microdilution, standardized methodology, *Mycoplasma hyorhinis*

## Abstract

Conducting antimicrobial susceptibility testing (AST) in a comparable manner requires the availability of a standardized method. Organizations, such as the Clinical and Laboratory Standards Institute (CLSI) or the European Committee on Antimicrobial Susceptibility Testing (EUCAST), provide standardized protocols for a range of fastidious bacteria but not for *Mycoplasma hyorhinis*. We developed a broth microdilution method for testing *M. hyorhinis* in a standardized and harmonized way using a modified Friis broth devoid of antimicrobial or otherwise bacterial growth-inhibiting agents. The type strain *M. hyorhinis* DSM 25591 was chosen to establish the methodology. The antimicrobial agents of interest were doxycycline, enrofloxacin, erythromycin, florfenicol, gentamicin, marbofloxacin, tetracycline, tiamulin, tilmicosin, tulathromycin, and tylosin, tested by using commercial Sensititre^TM^ microtiter plates. In addition, the suitability of the methodology was evaluated via variation of the individual ingredients of the modified Friis broth by either using different batches or choosing other distributors. Despite these alterations, the method provided reliable results. We obtained repeatable minimal inhibitory concentrations for all six tested field isolates and the *M. hyorhinis* type strain. With this newly proposed method, we aim to provide an improved AST method for diagnostic laboratories and monitoring purposes with better comparability between times and countries. In addition, this new method will allow for an improvement of targeted treatments using antimicrobial agents and thereby reduce the options for resistance development.

## 1. Introduction

*Mycoplasma* (*M.*) *hyorhinis* is a globally prevalent bacterium in pigs. For a long time, it was considered a commensal, inhabiting mucous membranes of the upper respiratory tract and only rarely causing disease [1,2]. Growing evidence as well as an increasing number of case reports indicate its emergence as an important cause of polyserositis and polyarthritis in nursery piglets at the age of 3–10 weeks, and other disease manifestations, such as conjunctivitis and meningitis in older pigs [1,2,3,4,5,6,7].

In general, mycoplasmas—as cell-wall-free organisms—are intrinsically resistant to all beta-lactams and other substances targeting the cell wall [8,9]. Moreover, the efficacy of antimicrobial agents targeting the folic acid metabolism (e.g., sulfonamides and trimethoprim) might be impaired since mycoplasmas mostly use exogenic folates provided by the host due to enzyme gaps within the genome concerning cofactor metabolism [10,11]. It is known that certain porcine mycoplasmas developed resistance against 14-membered macrolides, more specifically erythromycin, though the phenotypic manifestation is variable within field isolates [12]. The resistance is conferred by a point mutation within the 23S rRNA [13,14]. Correspondingly, the literature reports increased minimal inhibitory concentrations (MICs) for erythromycin among *M. hyorhinis* field isolates [6,8,9,15,16].

Apart from known resistances, it is important to monitor field isolates for their antimicrobial susceptibility to be aware of occurring and emerging resistances and changing susceptibility profiles. The choice of the right antimicrobial agent for treatment is usually based on the knowledge of occurring susceptibility profiles within the herd or population of bacteria. Treatment failure often occurs because of knowledge gaps as well as test failures due to a lack of standardization [17].

Published literature on antimicrobial susceptibility testing (AST) of *M. hyorhinis* is available but scarce. The interpretation of the data is complicated, and the results of different laboratories are not always comparable due to differing methodologies. In general, the use of a standardized AST methodology for clinical isolates results in comparable, and due to uniform quality control measures, valid data [17].

The Clinical and Laboratory Standards Institute (CLSI) published a document for standardized AST of human *Mycoplasma* and *Ureaplasma* spp. in 2011 [18]. So far, a standardized methodology for mycoplasmas of animal origin has not been established. Still, studies report increased MIC values for several antimicrobial substances and isolates of different origins or deem isolates susceptible or resistant [6,19,20].

Standardizing the methodology by generating repeatable data, using the type strain *M. hyorhinis* DSM 25591 as a prospective quality control strain, and establishing a broth microdilution method, we aim to provide laboratories with the possibility of generating comparable data in the future.

## 2. Materials and Methods

### 2.1. Bacterial Strains

The type strain of *M. hyorhinis* DSM 25591 (ATCC 17981) was purchased from the German Collection of Microorganisms and Cell Cultures GmbH (DSMZ, Braunschweig, Germany). For the purpose of broth media evaluation, the type strain of *M. hyopneumoniae* NCTC 10110 (ATCC 25934) was procured at the National Collection of Type Cultures (NCTC, Salisbury, UK). The strains *Enterococcus faecalis* DSM 2570 (ATCC 29212) and *Staphylococcus aureus* DSM 2569 (ATCC 29213) were obtained from the DSMZ as well and used as quality control strains in antimicrobial susceptibility testing, as previously described [21]. A total of nine field isolates of *M. hyorhinis* were derived from clinical cases (nasal cavity, lung, broncho-alveolar lavage fluid, joint, and serosa) collected over an 18-year period (2003–2021) in Austria and Germany (Table 1). All isolates were confirmed to be *M. hyorhinis* by a *Mycoplasma*-specific nested PCR and subsequent sequencing of the PCR product [22]. These field isolates served for the determination of growth curves and the development of the AST method.

### 2.2. Media

Since mycoplasmas are fastidious bacteria, the usual Mueller Hinton broth is not suitable as a culture or test medium. More complex broth media are required. The complex Friis broth, specifically developed for culturing *M. hyopneumoniae* by N. Friis in 1975, was chosen for evaluation [23]. The Friis broth is also recommended for the culture of *M. hyorhinis* by the DSMZ [24]. In this study, we modified the original Friis medium. The modifications were as follows: (i) To make sure that the bacteria survive the freeze-thaw cycle without adding other cryopreservatives, we increased the amount of porcine serum to at least 20%. (ii) For reasons of a better evaluation of the color change, the amount of phenol red solution was doubled. (iii) The volumes of the yeast extract solution and deionized water were also changed to maintain the equilibrium of the provided nutrients. This medium was referred to as modified Friis broth.

Approximately 176 mL of modified Friis broth are composed of 0.82 g of porcine Brain Heart Infusion (BHI) (Sigma-Aldrich Chemie GmbH, Taufkirchen, Germany), 0.87 g of Difco^TM^ Mycoplasma PPLO Broth w/o CV (Becton Dickinson (BD), NJ, USA), 50 mL of Hank’s Balanced Salt Solution (Gibco^®^, Thermo Fisher Scientific, Waltham, MA, USA), and 78 mL of deionized water. Before autoclaving (15 min at 121 °C, 1 bar), the pH was set to 7.4. After cooling, 40.6 mL of heat-inactivated porcine serum (Gibco^®^), 4.49 mL of 25% autoclaved yeast extract solution (Carl Roth GmbH + Co. KG, Karlsruhe Germany), 0.361 mL of 1% filter sterilized phenol red solution (phenol red sodium salt, Carl Roth GmbH; 22 µm ROTILABO^®^ MCE syringe filter, Carl Roth GmbH), and 0.285 mL of autoclaved deionized water were supplemented. For solid media, 1% Agar-Agar (Carl Roth GmbH) was added before autoclaving.

The modified Friis broth was used as a culture and test medium for the broth microdilution method, as previously described [21]. The stability of the broth as a test medium was evaluated by batch testing of individual substances. The main substances, namely BHI, PPLO broth base, and yeast extract solution, were individually exchanged. The following ingredients were used: (i) porcine BHI (BD), (ii) bovine BHI (Oxoid^TM^, Wesel, Germany); (iii) a second batch of Difco^TM^ Mycoplasma PPLO Broth w/o CV (BD), (iv) Mycoplasma broth base (Oxoid); (v) a second batch of yeast extract by Carl Roth GmbH, and (vi) another yeast extract supplied by Oxoid. The yeast extract needed to be autoclaved more carefully at 105 °C, 1 bar for 15 min. When autoclaving at a higher temperature, the yeast extract degenerates and precipitates. The inactivation of the porcine serum was carried out in a 56 °C water bath for 30 min. As the heat-inactivated porcine serum was rich in fatty aggregates, the inactivated serum was filtered (70 µm cell strainer, Sarstedt, Nümbrecht, Germany) and centrifuged (400× *g* for 2 min.). The clear filtrates were subsequently aliquoted and stored at −20 °C until needed.

The SP4 medium has been used for the cultivation of spiroplasmas and highly fastidious mycoplasmas; it has also been recommended in the CLSI document for AST of human mycoplasmas [18,25]. Therefore, the ability of this medium to promote the growth of porcine mycoplasmas was evaluated. The SP4 broth was prepared according to CLSI recommendations [18]. One hundred milliliters of SP4 broth are comprised of 64.3 mL deionized water, 0.35 g Mycoplasma broth base (Difco^TM^ Mycoplasma PPLO Broth w/o CV; BD, NJ, USA), 1 g of tryptone (Sigma-Aldrich Chemie GmbH), 0.53 g of peptone (peptone ex casein, Carl Roth GmbH), 0.2 mL of a 1% phenol red solution (phenol red sodium salt, Carl Roth GmbH) and 0.02 g DNA (DNA from fish sperm, SERVA Electrophoresis GmbH, Heidelberg, Germany). For the preparation of SP4 agar medium, 1.5 g of Agar-Agar (Carl Roth GmbH) was added. The broth base was autoclaved for 15 min at 121 °C, 1 bar. The following supplements were added: 5 mL of CMRL-1066 10X w/o L-glutamine (Gibco^®^), 3.5 mL of an autoclaved 25% yeast extract solution (Carl Roth GmbH, prepared as described above), 17 mL heat-inactivated fetal calf serum (Sigma-Aldrich Chemie GmbH), 10 mL of a 2% Yeastolate solution (Bacto^TM^, Gibco^®^) and 1 mL of a 50% Glucose solution (Carl Roth GmbH).

### 2.3. Growth Curves

At first, the suitability of the broth media was evaluated by setting up growth curves of the type strains *M. hyorhinis* DSM 25591 and *M. hyopneumoniae* NCTC 10110 in triplicate. Five mL of modified Friis or SP4 broth were inoculated with 1 × 10^4^ CFU/mL of *M. hyorhinis* or *M. hyopneumoniae*, incubated aerobically at 37 °C for seven (*M. hyorhinis*) or 14 days (*M. hyopneumoniae*), respectively. Every 24 h, 120 µL of broth cultures were serially diluted and plated onto the corresponding solid media. Then, agar plates were incubated at 37 °C under 5% CO_2_ atmosphere, and colonies were counted after seven days (*M. hyorhinis*) or 14 days (*M. hyopneumoniae*) of incubation.

In addition, seven field isolates of *M. hyorhinis* were chosen to evaluate their growth properties. The incubation of broth cultures was prolonged to ten days. Here, only the modified Friis broth was used. Again, 5 mL of modified Friis broth were inoculated with 1 × 10^4^ CFU/mL, 120 µL taken every 24 h, serially diluted, plated onto modified Friis agar, and colonies counted after incubation at 37 °C and 5% CO_2_ for three to ten days.

### 2.4. Storage and Quantification of Mycoplasma hyorhinis and M. hyopneumoniae

The *M. hyorhinis* field isolates, as well as the *M. hyorhinis* and *M. hyopneumoniae* type strains (DSM 25591, NCTC 10110), were cultured in modified Friis broth as described above. After a color change of the broth culture occurred, 500 µL were subcultured into 5 mL of freshly prepared modified Friis broth to ensure that the culture used for AST was metabolically active and within its log- or stationary phase. Finally, the subculture was incubated at 37 °C until a color change from red to yellow was observed, aliquoted, and stored at −80 °C until further used.

**Figure 1:**
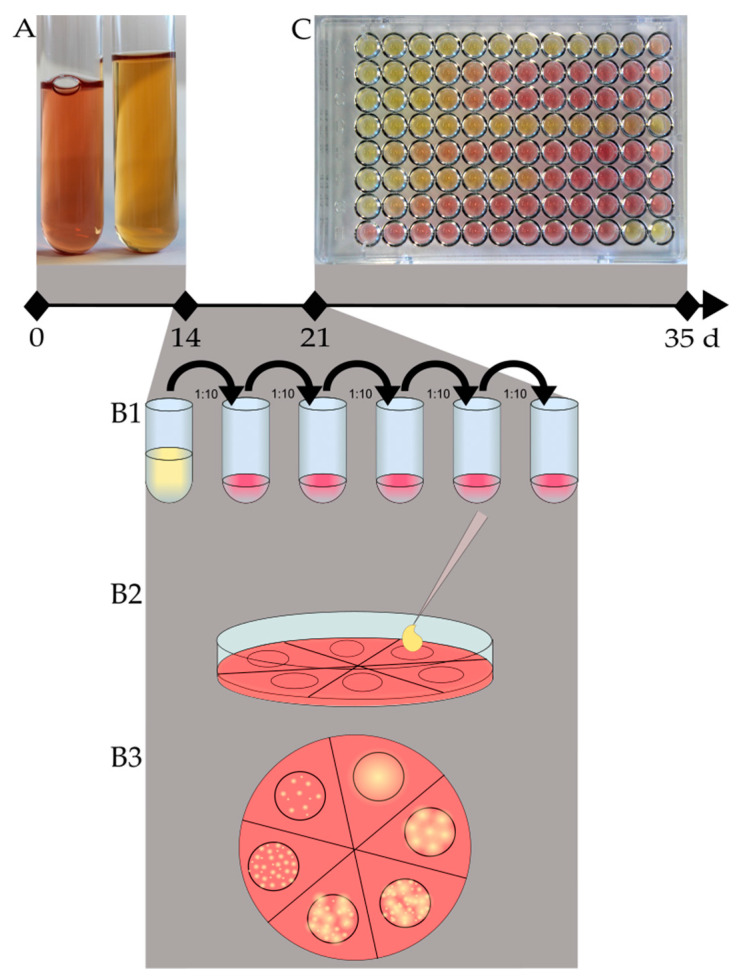
Schematic workflow and timeline for cultivation and antimicrobial susceptibility testing of *M. hyorhinis*. (**A**) According to CLSI, at least two subcultures of a properly grown culture are needed. Growth is characterized by a color change of the medium (pH shift) from red (no growth/growth control—left tube) to yellow (grown culture—right tube). (**B**) Cultures are serially diluted (tenfold) (**B1**) over five steps; 20 µL of each dilution step, as well as the original culture, are dropped onto a Friis agar plate (**B2**). The agar plate was divided into six sections. The area of the inoculum was encircled so that the area of growth can later be found easily. After incubation for up to 14 days, each section was evaluated for the presence of colonies using a stereo microscope. (**B3**) Sections with 30 to 300 single colonies were used for calculating the CFU/mL (**C**) Depicted is an example of a microtiter plate after the maximum time of incubation (14 days), inoculated with the type strain *M. hyorhinis* DSM 25591.

Before freezing, bacterial counts were determined for each culture according to CLSI by setting up a tenfold dilution series over five steps [18]. For this purpose, a modified Friis agar plate was divided into six sections, and 20 µL of each dilution step, as well as the original culture, were dropped onto the agar surface, air-dried, and incubated at 37 °C, 5% CO_2_ until individual colonies were detected using a stereo microscope (usually after 3 to 7 days for *M. hyorhinis*, and 10 to 14 days for *M. hyopneumoniae*). Cultures on agar plates without any detectable colonies after 14 and 21 days of incubation were considered negative. Agar sections containing 30 to 300 individual colonies were used for calculating the number of CFU/mL in the original broth culture (Figure 1).

This method of quantification was also used to ensure that the inoculum density was appropriate for AST. Since the expected CFU count for the inoculum density is comparably low, only two dilution steps were carried out.

### 2.5. Antimicrobial Susceptibility Testing of M. hyorhinis

The provisional use of the quality control strains *E. faecalis* ATCC 29212 and *S. aureus* ATCC 29213 for validation purposes of the establishment of the AST method was previously described in detail [21]. CLSI standards were applied, except for using modified Friis broth instead of cation-adjusted Mueller Hinton broth and extending the incubation and reading times due to prolonged incubation times of the *M. hyorhinis* inoculated microtiter plates.

The inoculum of the *M. hyorhinis* strains was set up based on the CLSI document describing AST of human mycoplasmas [18]. The targeted inoculum contained 1 × 10^5^ CFU/mL, with an accepted range between 5 × 10^4^ CFU/mL to 5 × 10^5^ CFU/mL. According to the respective colony count, the required volume of the frozen stock culture for inoculum preparation was assessed, and calculated volumes of freshly thawed stock cultures were transferred into pre-warmed (room temperature) modified Friis broth.

The inoculum suspensions were then pre-incubated for 2 h at 37 °C, 7.5% CO_2_. This pre-incubation step helped the mycoplasmas to regain viability and retrieve metabolic activity. This step is equivalent to a fresh overnight culture of non-fastidious bacteria used for AST.

Following the pre-incubation, 50 µL of the inoculum suspension were transferred into each well of the Sensititre^TM^ microtiter plates (Thermo Fisher Scientific), properly sealed with the provided adhesive foil to prevent evaporation and incubated at 37 °C and ambient air, until a color change of the medium, indicating bacterial growth, was observed in the growth control wells. The following antimicrobial agents were investigated: clindamycin (0.03–64 mg/L), doxycycline (0.06–128 mg/L), enrofloxacin (0.008–16 mg/L), erythromycin (0.015–32 mg/L), florfenicol (0.12–256 mg/L), gentamicin (0.12–256 mg/L), marbofloxacin (0.008–16 mg/L), tetracycline (0.12–256 mg/L), tiamulin (0.03–64 mg/L), tilmicosin (0.06–128 mg/L), tulathromycin (0.06–32 mg/L), and tylosin (0.06–128 mg/L). The microtiter plates used in these experiments corresponded to those used in the national resistance monitoring program GE*RM*-Vet [26].

**Figure 2:**
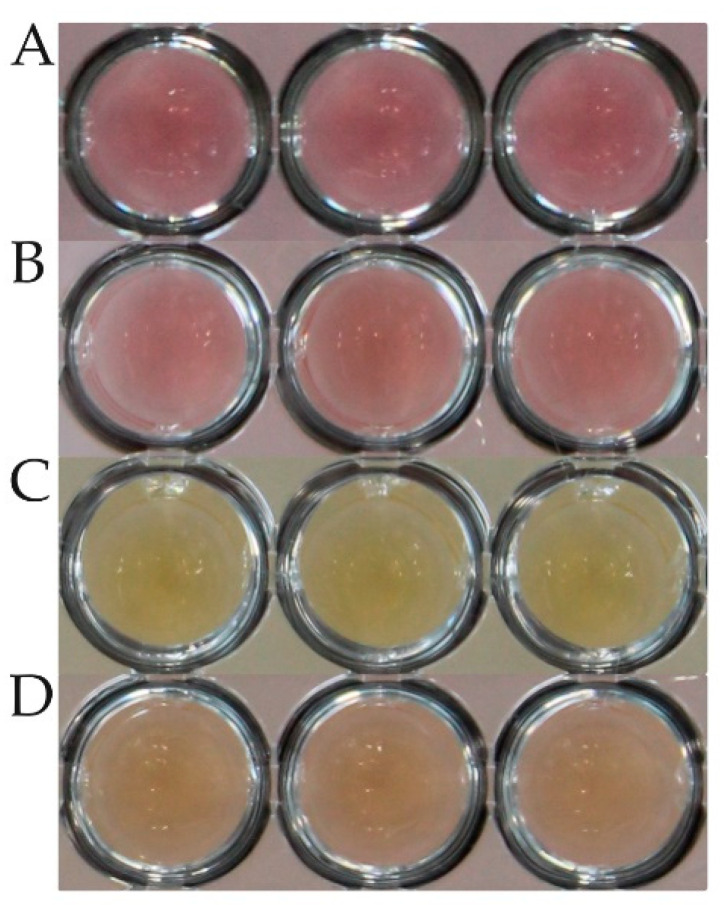
Color of inoculum in wells of a microtiter plate after 14 days of incubation. All plates were incubated at 37 °C and ambient air. (**A**) modified Friis broth without mycoplasmas (**B**) *M. hyorhinis* DSM 25591, no visible growth and no color change (red). (**C**) *M. hyorhinis* DSM 25591, visible growth indicated by complete color change (yellow). (**D**) *M. hyorhinis* DSM 25591, trailing with incomplete color change (orange).

The MICs of the quality control strains *E. faecalis* ATCC 29212 and *S. aureus* ATCC 29213 were recorded according to CLSI [27]. The MICs of *M. hyorhinis* strains were defined as the first well, where the color change of the medium was incomplete, meaning the shift from red to yellow did not occur. Trailing was observed (orange color), which was not defined as growth. During the entire experimental phase, the microtiter plates were inspected with the unaided eye by the same person. The best way to evaluate the color change was by gently lifting the microtiter plate and evaluating the color change from below. The microtiter plates were checked daily for color changes (Figure 2). Once the color change in the growth control wells was observed, the MICs were recorded. The plates were then further incubated for at least another 24 h, and MICs were recorded again. Only when the color change was complete, and no major changes (more than one dilution step difference compared to the prior MIC recording) of MIC values occurred, the reading of the plates was termed final.

## 3. Results & Discussion

### 3.1. Broth Media Comparison and Growth Curves

**Figure 3:**
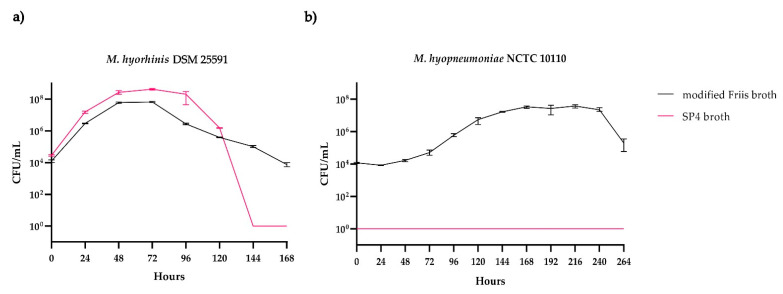
Growth curves of (**a**) *M. hyorhinis* DSM 25591 and (**b**) *M. hyopneumoniae* NCTC 10110 cultured in modified Friis broth (black) or SP4 broth (red). Both strains were cultured in triplicates. The cell count was determined on the corresponding agar media.

Cultures of *M. hyorhinis* DSM 25591 showed similar growth curves for both broth media tested (Figure 3a). The total numbers of CFU/mL in SP4 broth were higher than those in modified Friis broth. This can be partly explained by a slightly higher starting CFU/mL inoculated into SP4 broth compared to modified Friis broth. On the other hand, the viability of *M. hyorhinis* DSM 25591 seemed to be better supported by the modified Friis broth. The onset of the death phase in modified Friis broth was less sudden than in SP4 broth, in which the number of viable cells strongly decreased after four days of incubation. The doubling time of *M. hyorhinis* in modified Friis broth was calculated to be 3 h and 54 min. For cultures grown in SP4 broth, the doubling time was calculated to be 3 h and 38 min.

For *M. hyopneumoniae* NCTC 10110, growth was only observed in modified Friis broth (Figure 3b). Though the strain was cultured and sampled for 14 days, colonies on the modified Friis agar plates were only produced in samples taken until the 11th day (264 h) of incubation in modified Friis broth. No growth was observed in SP4 broth or on SP4 agar plates. However, samples taken from an SP4 broth culture (incubated for eight days) and plated onto modified Friis agar plates revealed equal numbers of CFU, indicating that *M. hyopneumoniae* remained viable but did not grow in SP4 broth. One reason why *M. hyopneumoniae* did not show any growth on the SP4 agar might be the higher percentage of agarose compared to the modified Friis agar plates. It is known that the increase in agarose might decrease the successful growth of *M. hyopneumoniae* on solid media [28]. The doubling time of *M. hyopneumoniae* cultured in modified Friis broth was calculated to be 10 h and 44 min.

Based on these results, all further experiments were performed with modified Friis broth only, as it promotes the growth of both porcine *Mycoplasma* species.

**Figure 4:**
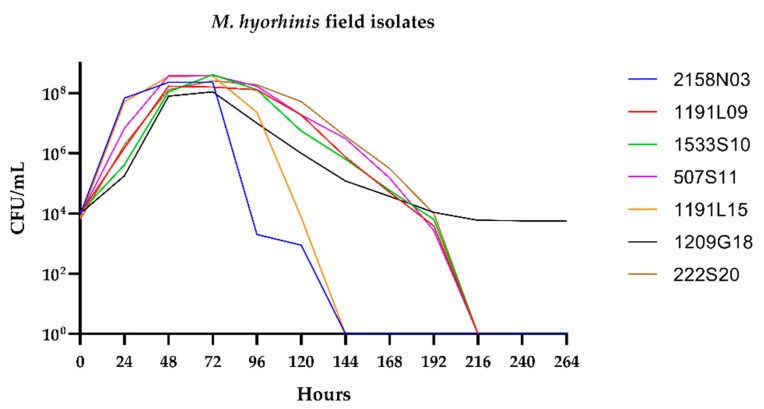
Growth curves of seven *M. hyorhinis* field isolates cultured in modified Friis broth for eleven days.

The growth curves of seven different *M. hyorhinis* field isolates were determined in modified Friis broth. The isolates showed variable growth rates (Figure 4), with doubling times ranging from 3 h and 5 min (1191L15) to 3 h and 42 min (1209G18). This was expected, as varying growth rates have been described for different *M. hyorhinis* isolates before [29,30]. The growth curves of the field isolates indicated that the planned reading times of the AST microtiter plates at fixed time points might not be feasible, and individual evaluation of the color change is needed.

The daily removal of 120 µL for CFU determination had no influence on the final CFU counts.

### 3.2. Antimicrobial Susceptibility Testing of M. hyorhinis DSM 25591

The twelve antimicrobial substances tested were chosen according to their therapeutic use in the field but also because of possible resistance developments against them [31]. The porcine mycoplasmas *M. hyorhinis* and *M. hyopneumoniae* are described to be susceptible to aminoglycosides, fluoroquinolones, phenicols, pleuromutilins, and tetracyclines, whereas the susceptibility to macrolides was shown to be variable [12]. Intrinsic resistance to beta-lactams as well as sulfonamides and trimethoprim is well known [10,32]. As representative of the class of lincosamides, the substance clindamycin was added as well [33].

Twenty-two susceptibility tests of *M. hyorhinis* DSM 25591 were conducted on independent occasions using the modified Friis broth as described above. The individual repetitions were performed to establish repeatable MICs of the type strain as a comparison for batch testing. The microtiter plates were evaluated daily. Whenever the growth controls showed a full-color change, the microtiter plates were evaluated. The color change was defined as the change from red (no growth) to yellow (growth). The final evaluation of the microtiter plates was marked by two readings 24 h apart, with no major change in MIC values (endpoint).

This resulted in MIC values mostly spanning two to three dilution steps (Table 2). In doxycycline (≤0.06 mg/L) and tetracycline (≤0.12 mg/L) containing wells, no growth was detected throughout all 22 tests. The MIC values for gentamicin (1–2 mg/L), enrofloxacin and marbofloxacin (0.5–1 mg/L), clindamycin (0.12–0.25 mg/L), tulathromycin (≤0.06–0.12 mg/L) and tiamulin (≤0.03–0.06 mg/L) clustered around two successive dilution steps. For gentamicin, this resulted in an equal distribution between 1 and 2 mg/L. Regarding enrofloxacin, marbofloxacin, clindamycin, tulathromycin, and tiamulin, the MIC values mostly clustered on one dilution step, with only a minor number of tests resulting in different MICs. MICs ranging over three dilution steps were noticed for the substances florfenicol (0.25–1 mg/L), tylosin (≤0.06–0.25 mg/L), and erythromycin (8–32 mg/L). It is noteworthy that the middle value always peaked, indicating a main MIC value ± one dilution step. Only for tilmicosin, the MICs ranged over four dilution steps (0.5–4 mg/L), although the lowest MIC of 0.5 mg/L was detected only once.

To assess the suitability of the modified Friis broth as test medium, the mode MIC was established based on the MIC values given in Table 2. The mode MIC describes the homogeneity of repeated AST results. Based on the degree of identity between individual tests, the exact and essential MIC agreement can be calculated. The exact MIC agreement describes the percentage of MIC values, matching exactly the mode MIC. The essential MIC agreement gives the percentage of MIC values matching the mode MIC, including one dilution step below and above (± one dilution step).

An exact MIC agreement was observed for the antimicrobial agents gentamicin, doxycycline, and tetracycline. For the class of tetracyclines, this meant consistently no growth. Gentamicin was equally distributed across two dilution steps, wherefore they were taken together as one mode MIC. The exact MIC agreement of clindamycin was calculated to be 91%, and for marbofloxacin and tiamulin, both 82%. The mode MIC of tulathromycin was exactly repeated in 73% of the tests, that of enrofloxacin in 64%, and that of tylosin in 55%. The lowest exact MIC agreements of 50% were calculated for florfenicol, erythromycin, and tilmicosin.

For all antimicrobial substances, except for tilmicosin, the essential MIC agreement was 100%. One test showed a lower MIC value of tilmicosin (−2 dilution steps from the mode MIC), resulting in an essential MIC agreement of 95%. According to CLSI standards, an essential agreement of ≥95% for a possible control strain is required [34]. Thus, all tested substances fall within this category (Table 3, far right column). The mean essential agreement was calculated to be 99.62%.

Since a deviation of one dilution step from the mode MIC is acceptable, the essential MIC agreement is of importance for evaluating a test method. For *M. hyorhinis* DSM 25591, together with the modified Friis broth, as applied here, the criterium given by the CLSI documents is fulfilled, as well as ISO Directive 20776-2:2021 [34,35]. Therefore, we conclude that the use of the modified Friis broth is an acceptable choice, not only as a culture medium but also as a test medium.

After determining a basic MIC distribution by individual repetitions of the susceptibility tests, the main ingredients of the modified Friis broth were individually exchanged. The six different settings described in the Material and Methods section were repeated three times, and the initial broth served as a control in each repetition (resulting in a total of 21 individual tests). For better comparability, the initial MIC ranges are highlighted in green (Table 4).

For most antimicrobial agents, the obtained MIC values for *M. hyorhinis* DSM 25591 fell within the expected ranges shown in Table 2. Only for gentamicin and tiamulin the MIC results exceeded one dilution step. For clindamycin, the batch testing resulted in an increase in two dilution steps (Table 4).

We again evaluated the percentage of deviating MIC values by calculating the MIC agreements (Table 5). The mode MIC was based on the initial tests of *M. hyorhinis* DSM 25591 in modified Friis broth as described above (Table 3).

The exact MIC agreements showed a greater variance than in the initial tests, ranging from the lowest exact agreement of 38% (tiamulin) to 100% (doxycycline and tetracycline). For most antimicrobial agents, the individual exchange of broth ingredients resulted in an essential MIC agreement of 95–100%. Only for tiamulin, the essential MIC agreement was lower, at 71% (Table 5).

By comparing the initial mode MICs (Table 3) with the batch comparison (Table 4), and the essential MIC agreements, respectively, it can be concluded that for most antimicrobial agents, the modified Friis broth is suitable. According to the CLSI document M23, the required essential MIC agreement of ≥95% for a type strain is met on all accounts except for tiamulin (Table 5, batch testing) [34]. The lower agreement for tiamulin could be in part due to the mode MIC, which was at the lowest tiamulin concentration in the microtiter plates. However, when considering the distribution of MIC values for tiamulin, all three MIC values are almost evenly distributed. If only one of the tests resulting in a MIC of ≤0.03 mg/L would have resulted in a one dilution step higher MIC, a mode MIC of 0.06 mg/L ± one dilution step would have been achieved, resulting in an essential agreement of 100% instead of 71%. When calculating the mean essential agreement for both datasets (initial tests: 99.62%; batch tests: 97.22%), it can be concluded that the influence of different medium ingredients within this complex broth medium is only of a minor nature.

### 3.3. Antimicrobial Susceptibility Testing of M. hyorhinis Field Isolates

Finally, we utilized a selection of six field isolates to validate the standardized method (Table 6). The field isolates were chosen according to the year of isolation as well as the source of isolation (country, organs). Although erythromycin resistance is fairly common among porcine mycoplasmas, we evaluated the susceptibility of *M. hyorhinis* towards erythromycin to monitor the occurrence of elevated MICs in field isolates and not to overlook any possible changes [12,13,14,36].

For the antimicrobial agents gentamicin, enrofloxacin, marbofloxacin, florfenicol, erythromycin, tiamulin, doxycycline, and tetracycline unimodal distributions of MIC values over two to three dilution steps were observed (Table 6). For the macrolides tilmicosin, tulathromycin, and tylosin, as well as the lincosamide clindamycin, a bimodal distribution was noted. For substances with bimodal distributions, very high MIC values were occasionally detected. It was also noted that the elevated MIC values corresponded to the same isolates. Further studies are required to investigate whether mutations or acquired macrolide/lincosamide resistance genes account for the high macrolide/lincosamide MICs in these isolates.

The mode MIC was then assessed for each field isolate individually by testing each isolate in triplicate using the initial modified Friis broth composition. The individual MICs obtained from each field isolate during the three tests are presented in Supplemental Tables S1–S6. Subsequently, all six field isolates were tested using the aforementioned six settings with individually exchanged ingredients (batch testing), which resulted in six values per field isolate (a total of 36 values for all field isolates), and the deviations from the mode MIC were determined. Based on the mode MIC, the exact and essential MIC agreements were calculated. For field isolates, the CLSI document M23 dictates an essential MIC agreement of ≥90% to evaluate whether a broth medium is suitable [34].

The exact MIC agreement ranged from 42% (gentamicin) to 92% (tilmicosin). The essential MIC agreement, however, ranged from 78% (gentamicin) to 100% (enrofloxacin, marbofloxacin, clindamycin, tiamulin, doxycycline, tetracycline), with a mean essential agreement of 96.3% (Table 7).

With mean essential agreements of 99.62% (*M. hyorhinis* DSM 25591 testing/Table 3), 97.22% (*M. hyorhinis* DSM 25591 batch testing/Table 5), and 96.3% (field isolates of *M. hyorhinis*, batch testing/Table 7), the modified Friis broth, as presented here, proved to be a suitable culture and test medium.

Since mycoplasmas are generally very demanding concerning their culture conditions, it is necessary to accept certain difficulties, such as expensive or very complex broth media as well as the extended growth periods, also for AST methods. Several very different agar and broth media have been used over time for AST of porcine mycoplasmas: Friis media [6,8,9,25], modified Friis media [15,25,37], the commercial Liquid Mycoplasma Medium (Mycoplasma Experience Ltd., UK) [19,20,38], variations of YUS and CMRL media [30], M media [14,36,39] and KM2 media [40]. According to the published literature, for *M. hyorhinis* and *M. hyopneumoniae,* the (modified) Friis broth appears to be the most reliable culture medium so far. The CLSI published a guideline for AST of human mycoplasmas and ureaplasmas in 2011 [18]. Therein, a selection of *Mycoplasma*- and *Ureaplasma*-propagating broth media used for AST was listed. One of the employed media, the SP4 broth, was also recommended for animal mycoplasmas by Hannan (2000). This was the reason why the SP4 broth was included in our initial tests, to evaluate its suitability for *M. hyorhinis* and *M. hyopneumoniae* [25]. Since our aim was to provide a broth medium for both porcine mycoplasmas, the SP4 broth was not considered, as it did not promote the growth of *M. hyopneumoniae* NCTC 10110.

Due to the lack of a standardized methodology, the definition of clinical breakpoints and epidemiological cut-off values has not been accomplished for animal mycoplasmas so far. Therefore, it is not possible to classify field isolates as susceptible or resistant based on the generated MIC values. Ter Laak et al. (1991) and Hannan et al. (1997) suggested breakpoints for several *Mycoplasma* species, and subsequent studies usually compared the obtained MIC values with the given data in general [6,8,41]. The determination of the actual MIC is difficult, and since almost all mycoplasmas grow without visual turbidity as known from other bacteria, a pH indicator is used. Since *M. hyorhinis* and *M. hyopneumoniae* are glucose-fermenting mycoplasmas, medium-acidifying metabolites of glycolysis are indicative of growth. Hannan (2000) already stated over 20 years ago that the color change for mycoplasmas with glycolytic activity might be something between orange and yellow [25]. We further defined the color change as growth (yellow), no growth (red), and trailing (orange), the latter of which was finally categorized as no growth but marked for observational purposes (Figure 1). The trailing phenomenon is also described, e.g., for enterococci tested with chloramphenicol, erythromycin, or tetracycline in CLSI documents [42].

Inter-laboratory comparisons are needed to evaluate potential disagreements between different persons conducting the tests with the same *Mycoplasma* isolates. The interpretation of a color change is, next to the age of the substances used in the broth, also dependent on the surrounding light conditions. Admittedly, the evaluation is, in part, a more subjective measurement, especially when evaluating trailing (Figure 2). A solution to the individual bias in evaluating color changes could be a photometric assay that measures the absorbance at specified wavelengths. The determination of ranges of absorbance that define growth, no growth, and trailing might be a further step in harmonization of this method and making it better reproducible in other laboratories. However, such an approach would be a study on its own in which different wavelengths, different spectrophotometers, and other parameters need to be compared.

The mode MIC deviations of tiamulin in the batch testing data set are mainly due to the fact that the mode MIC falls on the lowest MIC within the observed MICs (Table 5). This is most unfortunate since, for evaluation purposes, the dilution range should always cover at least one additional dilution step above and below the mode MIC. During the initial tests, this requirement held true. We argue that the MIC agreement for tiamulin within the batch testing is, therefore, slightly skewed, leading to a lower essential agreement percentage, though still falling within the acceptable spanning range of a maximum of three to four dilution steps in total. In general, tiamulin is stable under neutral and acid conditions [43]. The differing protein and electrolyte content within the different media components, however, might slightly influence the efficacy of tiamulin in our setting, explaining the slight difference in the resulting MIC values.

For the decreased essential MIC agreement of the field isolates testing with gentamicin, we consider the low number of tested isolates as a possible factor. The replicates of the type strain *M. hyorhinis* DSM 25591 gave solid results within two to three dilution steps, whereas the MIC values of repeated testing resulted in a 50-50 distribution; only the individual ingredient exchange resulted in the spread over three dilution steps. It is described that aminoglycosides might be affected by the change of e. g., pH or cations in the medium [44]. The observed deviations were not specific to one of the exchanged ingredients. Each of the six described settings was represented at least once among the deviating results.

Although the AST methodology previously used was different between working groups, most still agree on using the type strain of *M. hyorhinis* as a reference and for quality control purposes without generally valid quality control ranges [8,9,14,15,16,19,25,36,37,40]. Due to the variety of broth media used in broth microdilution methods, the resulting MIC values might differ as well. Furthermore, the incubation times differ between studies. Most agree on evaluating the microtiter plates on a daily basis until a color change occurs, whereas others read the plates at more or less fixed time points from up to two to seven days of incubation [8,9,15,25,36,40].

We noticed that for some of the tested antimicrobial agents, such as tulathromycin, tylosin, tiamulin, doxycycline, and tetracycline, the MICs were at the lower end of the test range. Thus, we suggest that specifically designed microtiter plate layouts should be used in future AST of *M. hyorhinis*, which contain even lower test concentrations for these antimicrobial agents. Such microtiter plates are currently not commercially available but will enable the determination of the lower endpoints of MICs of the aforementioned five antimicrobial agents.

Finally, enumerating mycoplasmas to properly determine the correct inoculum size requires a special methodology. Since liquid cultures lack turbidity, the usually applied McFarland standard, utilizing optical density, is not applicable for mycoplasmas. For this, the method of determining color-changing units (CCU) was established. This method describes a tenfold dilution titration, where the last dilution step, when a color change can still be seen, accounts for the CCU/mL. According to Hannan (2000), CCU and colony-forming units (CFU) can be used synonymously [25]. Since *M. hyorhinis* grows on Friis agar plates, we decided to prepare the inoculum by enumerating the CFU/mL of a grown culture and adjusting the inoculum to a defined bacterial content. In doing so, the inocula were standardized and reproducible.

Although this list of deviations in the methodologies does not mean to be complete, it represents a variety of methodological aspects, which makes it impossible to compare the so far published data. By presenting a standardized way of determining antimicrobial susceptibility data, we hope to reduce the lack of comparability in the future. In agreement with the published literature, the type strain of *M. hyorhinis* (e.g., DSM 25591, ATCC 17981, NCTC 10130) provided repeatable results and, thus, might be proposed as a future quality control strain.

## 4. Conclusions

Surveillance and monitoring of porcine pathogens, antimicrobial usage in the field, and antimicrobial resistance development are key factors in generating knowledge of the status quo and changes in phenotypic antimicrobial resistances. The constant development and establishment of improved methodologies and references, also for AST, is therefore one of the key aspects for systematic monitoring purposes.

Here, we presented a step towards a harmonized AST method for *M. hyorhinis* using the broth microdilution method with a standardized protocol of inoculum preparation and AST methodology. In addition, we propose the type strain of *M. hyorhinis* to be used as a quality control strain since this strain not only fulfills the requirements proposed by the CLSI and other associations (EUCAST, ISO) but also presented itself consistently throughout the investigation.

## Figures and Tables

**Table 1:** Origins of type strains and field isolates used for growth curves and the development of the AST method.

Isolate ID	Origin			Used in	
	Country	Year	Tissue	Growth Curves	AST
*M. hyorhinis* DSM 25591 type strain	Unknown	1955	Nasal cavity	x	x
*M. hyopneumoniae* NCTC 10110 type strain	Great Britain	1967	Nasal cavity	x	
*M. hyorhinis* 2158N03	Austria	2003	Nasal cavity	x	x
*M. hyorhinis* 1191L09	Austria	2009	Lung	x	x
*M. hyorhinis* 1533S10	Austria	2010	Serosa	x	
*M. hyorhinis* 507S11	Austria	2011	Serosa	x	x
*M. hyorhinis* 3661N14	Austria	2014	Nasal cavity		x
*M. hyorhinis* 1191L15	Austria	2015	Lung	x	
*M. hyorhinis* 1209G18	Austria	2018	Joint	x	
*M. hyorhinis* 222S20	Austria	2020	Serosa	x	x
*M. hyorhinis* T/0423263	Germany	2021	Lung (BALF)		x

**Table 2:** Distribution of the MIC values of *M. hyorhinis* DSM 25591 obtained in 22 individual tests.

Number of Tests and MIC Values Obtained (mg/L) *																	
Antimicrobial Agent	0.008	0.015	0.03	0.06	0.12	0.25	0.5	1	2	4	8	16	32	64	128	256	512
Gentamicin					-	-	-	11	11	-	-	-	-	-	-	-	-
Enrofloxacin	-	-	-	-	-	-	8	14	-	-	-	-	-				
Marbofloxacin	-	-	-	-	-	-	2	16	-	-	-	-	-				
Florfenicol					-	3	18	1	-	-	-	-	-	-	-	-	-
Clindamycin			-	-	2	20	-	-	-	-	-	-	-	-	-		
Erythromycin		-	-	-	-	-	-	-	-	-	3	11	8	-			
Tilmicosin				-	-	-	1	5	11	5	-	-	-	-	-	-	
Tulathromycin				16	6	-	-	-	-	-	-	-	-	-			
Tylosin				6	12	4	-	-	-	-	-	-	-	-	-	-	
Tiamulin			18	4	-	-	-	-	-	-	-	-	-	-	-		
Doxycycline				22	-	-	-	-	-	-	-	-	-	-	-	-	
Tetracycline					22	-	-	-	-	-	-	-	-	-	-	-	-

* Concentrations not included within the test panels are depicted as gray-shaded areas. When no color change was visible, the MIC value was set as equal to or lower than the lowest test concentration.

**Table 3:** Homogeneity of the MIC values of the 22 test replicates of *M. hyorhinis* DSM 25591, as given in Table 2. The grey shaded area describes the MIC values accounting for the essential MIC agreement.

	Deviation from Mode MIC							Exact MIC Agreement (%)	Essential MIC Agreement (%)
Antimicrobial Agent	−3	−2	−1	0	+1	+2	+3		
Gentamicin				22				100	100
Enrofloxacin			8	14				64	100
Marbofloxacin			3	18	1			82	100
Florfenicol			10	11	1			50	100
Clindamycin			2	20				91	100
Erythromycin			3	11	8			50	100
Tilmicosin		1	5	11	5			50	95
Tulathromycin				16	6			73	100
Tylosin			6	12	4			55	100
Tiamulin				18	4			82	100
Doxycycline				22				100	100
Tetracycline				22				100	100

**Table 4:** Distribution of the MIC values for *M. hyorhinis* DSM 25591. Twenty-one individual test results containing all tested batches and the respective controls are shown. For reasons of better comparability, the initial ranges of MIC results, given in Table 2, are highlighted in green.

Number of Tests and MIC Values Obtained (mg/L) *																	
Antimicrobial Agent	0.008	0.015	0.03	0.06	0.12	0.25	0.5	1	2	4	8	16	32	64	128	256	512
Gentamicin					-	-	-	4	12	5	-	-	-	-	-	-	-
Enrofloxacin	-	-	-	-	-	-	6	15	-	-	-	-	-				
Marbofloxacin	-	-	-	-	-	-	3	18	-	-	-	-	-				
Florfenicol					-	2	10	9	-	-	-	-	-	-	-	-	-
Clindamycin			-	-	3	9	8	1	-	-	-	-	-	-	-		
Erythromycin		-	-	-	-	-	-	-	-	-	2	10	9	-			
Tilmicosin				-	-	-	-	3	11	7	-	-	-	-	-	-	
Tulathromycin				15	6	-	-	-	-	-	-	-	-	-			
Tylosin				5	9	7	-	-	-	-	-	-	-	-	-	-	
Tiamulin			8	7	6	-	-	-	-	-	-	-	-	-	-		
Doxycycline				21	-	-	-	-	-	-	-	-	-	-	-	-	
Tetracycline					21	-	-	-	-	-	-	-	-	-	-	-	-

* Concentrations not included within the test panels are depicted as gray areas. When no color change was visible, the MIC value was set as equal to or lower than the lowest test concentration.

**Table 5:** Homogeneity of the MIC values of 21 test replicates of *M. hyorhinis* DSM 25591 tested in modified Friis broth, prepared with different batches of ingredients. The mode MIC and its deviations were determined based on the results of the initial testing of *M. hyorhinis* DSM 25591 (see Table 2). The grey shaded area contains the MIC values accounting for the essential MIC agreement.

	Deviation from Mode MIC							Exact MIC Agreement (%)	Essential MIC Agreement (%)
Antimicrobial Agent	−3	−2	−1	0	+1	+2	+3		
Gentamicin				16	5			76	100
Enrofloxacin			6	15				71	100
Marbofloxacin			3	18				86	100
Florfenicol			2	10	9			48	100
Clindamycin			3	9	8	1		43	95
Erythromycin			2	10	9			48	100
Tilmicosin			3	11	7			52	100
Tulathromycin				15	6			71	100
Tylosin			5	9	7			43	100
Tiamulin				8	7	6		38	71
Doxycycline				21				100	100
Tetracycline				21				100	100

**Table 6:** Distribution of the MIC values of six *M. hyorhinis* field isolates using the modified Friis broth. The MIC values represent the mode MICs of three independent tests of each isolate.

Number of Tests and MIC Values Obtained (mg/L) *																	
Antimicrobial Agent	0.008	0.015	0.03	0.06	0.12	0.25	0.5	1	2	4	8	16	32	64	128	256	512
Gentamicin					-	-	4	2	-	-	-	-	-	-	-	-	-
Enrofloxacin	-	-	-	-	-	-	2	2	2	-	-	-	-				
Marbofloxacin	-	-	-	-	-	-	-	4	2	-	-	-	-				
Florfenicol					-	2	3	1	-	-	-	-	-	-	-	-	-
Clindamycin			-	-	-	1	-	-	-	-	-	-	2	3	-		
Erythromycin		-	-	-	-	-	-	-	-	-	-	-	1	5			
Tilmicosin				-	-	-	-	-	1	-	-	-	-	-	-	5	
Tulathromycin				-	1	-	-	-	-	-	-	1	-	4			
Tylosin				-	-	1	-	-	-	-	-	-	1	4	-	-	
Tiamulin			-	1	3	2	-	-	-	-	-	-	-	-	-		
Doxycycline				-	-	4	2	-	-	-	-	-	-	-	-	-	
Tetracycline					2	2	2	-	-	-	-	-	-	-	-	-	-

* Concentrations not included within the test panels are depicted as gray-shaded areas. When no color change was visible, the MIC value was set as equal to or lower than the lowest test concentration. If growth was visible in all tested concentrations, the result was set as equal or higher than the next serially higher MIC value (counts shown as white numbers within grey-shaded areas).

**Table 7:** Homogeneity of the MIC values of six *M. hyorhinis* field isolates tested in modified Friis broth, prepared with different batches of ingredients. The mode MIC and its deviations were determined, based on the results of the initial testing of the isolates (see Table 6). The grey-shaded area contains the MIC values accounting for the essential MIC agreement.

	Deviation from Mode MIC							Exact MIC Agreement (%)	Essential MIC Agreement (%)
Antimicrobial Agent	−3	−2	−1	0	+1	+2	+3		
Gentamicin				15	13	6	2	42	78
Enrofloxacin			7	24	5			67	100
Marbofloxacin			2	25	9			69	100
Florfenicol		1	9	18	8			50	97
Clindamycin			4	31	1			86	100
Erythromycin		1	3	32				89	97
Tilmicosin	1		2	33				92	97
Tulathromycin			4	27	3	2		75	94
Tylosin		3	9	19	5			53	92
Tiamulin			3	26	7			72	100
Doxycycline			12	23	1			64	100
Tetracycline			8	19	9			53	100

## Data Availability

All data are contained within the article.

## Supplementary Materials

The following supporting information can be downloaded at: https://www.mdpi.com/article/10.3390/microorganisms11040994/s1, Table S1: Distribution of MICs of three independent tests of the *M. hyorhinis* field isolate 2158N03; Table S2: Distribution of MICs of three independent tests of the *M. hyorhinis* field isolate 507S11; Table S3: Distribution of MICs of three independent tests of the *M. hyorhinis* field isolate 1191L15; Table S4: Distribution of MICs of three independent tests of the *M. hyorhinis* field isolate 222S20; Table S5: Distribution of MICs of three independent tests of the *M. hyorhinis* field isolate 3661N14; Table S6: Distribution of MICs of three independent tests of the *M. hyorhinis* field isolate T/0423263.
