# Supplementary material for: Towards a Standardized Antimicrobial Susceptibility Testing Method for Mycoplasma hyorhinis
Source: Microorganisms. 2023 Apr 11;11(4):994. doi: 10.3390/microorganisms11040994 (PMC10140863; doi:10.3390/microorganisms11040994)
Supplement: Supplementary file 1 [file microorganisms-11-00994-s001.zip › microorganisms-2296963-supplementary.pdf]

Supplemental Tables, showing the individual minimal inhibitory concentrations (MICs) of the six *M. hyorhinis* field isolates used for the establishment of a proposed harmonized antimicrobial susceptibility testing protocol using the broth microdilution method. All isolates were tested using the modified Friis broth as described in *Material and Methods*.

Supplemental Table S1. Distribution of MICs of three independent tests of the *M. hyorhinis* field isolate 2158N03.

	**Number of tests and MIC values obtained (mg/L) ***																
**Antimicrobial agent**	**0.008**	**0.015**	**0.03**	**0.06**	**0.12**	**0.25**	**0.5**	**1**	**2**	**4**	**8**	**16**	**32**	**64**	**128**	**256**	**512**
Gentamicin					-	-	2	-	1	-	-	-	-	-	-	-	-
Enrofloxacin	-	-	-	-	-	-	1	1	1	-	-	-	-				
Marbofloxacin	-	-	-	-	-	-	-	2	1	-	-	-	-				
Florfenicol					-	-	2	1	-	-	-	-	-	-	-	-	-
Clindamycin			-	-	-	-	-	-	-	-	-	-	1	2	-		
Erythromycin		-	-	-	-	-	-	-	-	-	-	-	-	3			
Tilmicosin				-	-	-	-	-	-	-	-	-	-	-	-	3	
Tulathromycin				-	-	-	-	-	-	-	-	-	1	2			
Tylosin				-	-	-	-	-	-	-	-	-	-	3	-	-	
Tiamulin			-	-	-	3	-	-	-	-	-	-	-	-	-		
Doxycycline				-	1	2	-	-	-	-	-	-	-	-	-	-	
Tetracycline					2	1	-	-	-	-	-	-	-	-	-	-	-

* Concentrations not included within the test panels are depicted as gray-shaded areas. When no color change was visible, the MIC value was set as equal or lower than the lowest test concentration. If growth was visible in all tested concentrations, the result was set as equal or higher than the next serially higher MIC value (counts shown as white numbers within grey-shaded areas).

Supplemental Table S2. Distribution of MICs of three independent tests of the *M. hyorhinis* field isolate 507S11.

	**Number of tests and MIC values obtained (mg/L) ***																
**Antimicrobial agent**	**0.008**	**0.015**	**0.03**	**0.06**	**0.12**	**0.25**	**0.5**	**1**	**2**	**4**	**8**	**16**	**32**	**64**	**128**	**256**	**512**
Gentamicin					-	-	1	2	-	-	-	-	-	-	-	-	-
Enrofloxacin	-	-	-	-	-	-	-	1	2	-	-	-	-				
Marbofloxacin	-	-	-	-	-	-	-	-	3	-	-	-	-				
Florfenicol					-	-	3	-	-	-	-	-	-	-	-	-	-
Clindamycin			-	-	-	-	-	-	-	-	-	-	1	2	-		
Erythromycin		-	-	-	-	-	-	-	-	-	-	-	-	3			
Tilmicosin				-	-	-	-	-	-	-	-	-	-	-	-	3	
Tulathromycin				-	-	-	-	-	-	-	-	-	1	2			
Tylosin				-	-	-	-	-	-	-	-	-	1	2	-	-	
Tiamulin			-	-	1	2	-	-	-	-	-	-	-	-	-		
Doxycycline				-	-	-	3	-	-	-	-	-	-	-	-	-	
Tetracycline					-	-	3	-	-	-	-	-	-	-	-	-	-

* Concentrations not included within the test panels are depicted as gray-shaded areas. If growth was visible in all tested concentrations, the result was set as equal or higher than the next serially higher MIC value (counts shown as white numbers within grey-shaded areas).

Supplemental Table S3. Distribution of MICs of three independent tests of the *M. hyorhinis* field isolate 1191L15.

	Number of tests and MIC values obtained (mg/L) *																
**Antimicrobial agent**	**0.008**	**0.015**	**0.03**	**0.06**	**0.12**	**0.25**	**0.5**	**1**	**2**	**4**	**8**	**16**	**32**	**64**	**128**	**256**	**512**
Gentamicin					-	-	2	-	1	-	-	-	-	-	-	-	-
Enrofloxacin	-	-	-	-	-	-	-	3	-	-	-	-	-				
Marbofloxacin	-	-	-	-	-	-	-	2	1	-	-	-	-				
Florfenicol					-	-	1	2	-	-	-	-	-	-	-	-	-
Clindamycin			-	-	-	-	-	-	-	-	-	-	1	2	-		
Erythromycin		-	-	-	-	-	-	-	-	-	-	-	-	3			
Tilmicosin				-	-	-	-	-	-	-	-	-	-	-	-	3	
Tulathromycin				-	-	-	-	-	-	-	-	-	-	3			
Tylosin				-	-	-	-	-	-	-	-	-	-	3	-	-	
Tiamulin			-	-	2	1	-	-	-	-	-	-	-	-	-		
Doxycycline				-	-	1	2	-	-	-	-	-	-	-	-	-	
Tetracycline					-	-	3	-	-	-	-	-	-	-	-	-	-

* Concentrations not included within the test panels are depicted as gray-shaded areas. If growth was visible in all tested concentrations, the result was set as equal or higher than the next serially higher MIC value (counts shown as white numbers within grey-shaded areas).

Supplemental Table S4. Distribution of MICs of three independent tests of the *M. hyorhinis* field isolate 222S20.

	Number of tests and MIC values obtained (mg/L) *																
**Antimicrobial agent**	**0.008**	**0.015**	**0.03**	**0.06**	**0.12**	**0.25**	**0.5**	**1**	**2**	**4**	**8**	**16**	**32**	**64**	**128**	**256**	**512**
Gentamicin					-	-	-	3	-	-	-	-	-	-	-	-	-
Enrofloxacin	-	-	-	-	-	-	-	1	2	-	-	-	-				
Marbofloxacin	-	-	-	-	-	-	-	-	2	1	-	-	-				
Florfenicol					-	2	1	-	-	-	-	-	-	-	-	-	-
Clindamycin			-	-	-	3	-	-	-	-	-	-	-	-	-		
Erythromycin		-	-	-	-	-	-	-	-	-	-	1	2	-			
Tilmicosin				-	-	-	-	-	3	-	-	-	-	-	-	-	
Tulathromycin				-	2	1	-	-	-	-	-	-	-	-			
Tylosin				-	-	2	1	-	-	-	-	-	-	-	-	-	
Tiamulin			-	3	-	-	-	-	-	-	-	-	-	-	-		
Doxycycline				-	-	3	-	-	-	-	-	-	-	-	-	-	
Tetracycline					2	1	-	-	-	-	-	-	-	-	-	-	-

* Concentrations not included within the test panels are depicted as gray-shaded areas. When no color change was visible, the MIC value was set as equal or lower than the lowest test concentration.

Supplemental Table S5. Distribution of MICs of three independent tests of the *M. hyorhinis* field isolate 3661N14.

	Number of tests and MIC values obtained (mg/L) *																
**Antimicrobial agent**	**0.008**	**0.015**	**0.03**	**0.06**	**0.12**	**0.25**	**0.5**	**1**	**2**	**4**	**8**	**16**	**32**	**64**	**128**	**256**	**512**
Gentamicin					-	-	2	-	1	-	-	-	-	-	-	-	-
Enrofloxacin	-	-	-	-	-	-	2	1	-	-	-	-	-				
Marbofloxacin	-	-	-	-	-	-	-	3	-	-	-	-	-				
Florfenicol					-	-	3	-	-	-	-	-	-	-	-	-	-
Clindamycin			-	-	-	-	-	-	-	-	-	-	2	1	-		
Erythromycin		-	-	-	-	-	-	-	-	-	-	-	-	3			
Tilmicosin				-	-	-	-	-	-	-	-	-	-	-	-	3	
Tulathromycin				-	-	-	-	-	-	-	-	-	-	3			
Tylosin				-	-	-	-	-	-	-	-	-	1	2	-	-	
Tiamulin			-	-	2	1	-	-	-	-	-	-	-	-	-		
Doxycycline				-	-	3	-	-	-	-	-	-	-	-	-	-	
Tetracycline					-	2	1	-	-	-	-	-	-	-	-	-	-

* Concentrations not included within the test panels are depicted as gray-shaded areas. If growth was visible in all tested concentrations, the result was set as equal or higher than the next serially higher MIC value (counts shown as white numbers within grey-shaded areas).

Supplemental Table S6. Distribution of MICs of three independent tests of the *M. hyorhinis* field isolate T/0423263.

	Number of tests and MIC values obtained (mg/L) *																
**Antimicrobial agent**	**0.008**	**0.015**	**0.03**	**0.06**	**0.12**	**0.25**	**0.5**	**1**	**2**	**4**	**8**	**16**	**32**	**64**	**128**	**256**	**512**
Gentamicin					-	1	2	-	-	-	-	-	-	-	-	-	-
Enrofloxacin	-	-	-	-	-	-	3	-	-	-	-	-	-				
Marbofloxacin	-	-	-	-	-	-	-	3	-	-	-	-	-				
Florfenicol					1	2	-	-	-	-	-	-	-	-	-	-	-
Clindamycin			-	-	-	-	-	-	-	-	-	-	3	-	-		
Erythromycin		-	-	-	-	-	-	-	-	-	-	-	-	3			
Tilmicosin				-	-	-	-	-	-	-	-	-	-	-	1	2	
Tulathromycin				-	-	-	-	-	-	-	1	2	-	-			
Tylosin				-	-	-	-	-	-	-	-	-	3	-	-	-	
Tiamulin			-	1	2	-	-	-	-	-	-	-	-	-	-		
Doxycycline				-	1	2	-	-	-	-	-	-	-	-	-	-	
Tetracycline					1	2	-	-	-	-	-	-	-	-	-	-	-

* Concentrations not included within the test panels are depicted as gray-shaded areas. When no color change was visible, the MIC value was set as equal or lower than the lowest test concentration. If growth was visible in all tested concentrations, the result was set as equal or higher than the next serially higher MIC value (counts shown as white numbers within grey-shaded areas).
